# Antecolic reconstruction is associated with a lower incidence of delayed gastric emptying compared to retrocolic technique after Whipple or pylorus-preserving pancreaticoduodenectomy

**DOI:** 10.1097/MD.0000000000016663

**Published:** 2019-08-23

**Authors:** Jianguo Qiu, Ming Li, Chengyou Du

**Affiliations:** Department of Hepatobiliary Surgery, The First Affiliated Hospital of Chongqing Medical University, Chongqing, China.

**Keywords:** delayed gastric emptying, duodenojejunostomy, gastrojejunostomy, pancreaticoduodenectomy, pylorus-preserving pancreaticoduodenectomy

## Abstract

**Background:**

The aim of present study is to investigate the relationship between the antecolic (AC) route of gastrojejunostomy (GJ) after pancreaticoduodenectomy (PD) or duodenojejunostomy (DJ) reconstruction after pylorus-preserving pancreaticoduodenectomy (PPPD), and the incidence of delayed gastric emptying (DGE).

**Methods:**

An electronic search of 4 databases to identify all articles comparing AC and retrocolic (RC) reconstruction after PD or PPPD was performed.

**Results:**

Fifteen studies involving 2270 patients were included for final pooled analysis. The overall incidence of DGE was 27.2%. Meta-analysis results showed AC group had lower incidence of DGE (odds ratio, 0.29; 95% confidence interval [CI], 0.16–0.52, *P* < .0001) and shorter hospital length of stay (weight mean difference, −3.29; 95% CI, −5.2 to −1.39, *P* = .0007). Days until to liquid and solid diet in the AC group were also significantly earlier than that in the RC group (*P* = .0006 and *P* < .0001). There was no difference in operative time, incidence of pancreatic fistula and bile leakage, and mortality, respectively.

**Conclusions:**

AC route of GJ after PD or DJ after PPPD is associated with a lower incidence of DGE. However, the preferred route for GJ or DJ reconstruction remains to be investigated in well-powered, randomized, controlled trial.

## Abstract 2

Supplemental Digital Content is available in the text

## 1 Introduction

Pancreatoduodenectomy (PD) is the standard surgical treatment for pancreatic and other periampullary malignancies.^[1–3]^ Pylorus-preserving PD (PPPD) is a procedure with equal short- and long-term outcomes compared to the classic PD, which has been demonstrated by several studies.^[4–7]^ Both PD and PPPD procedures were considered the only possibly curative treatment. Despite the mortality of such procedures have decreased to less than 5% in high-volume surgical institutions, but postoperative morbidity remains relatively high.^[8]^ One of the most frequent postoperative complications after PD or PPPD is delayed gastric emptying (DGE), which ranges from 5% to 80% among published studies.^[9–13]^

Generally, DGE is not a lethal complication; it is associated with significantly longer hospital stay and higher costs. Several retrospective comparative studies (RSCSs), as well as prospective randomized trials (RCTs) comparing antecolic (AC) versus classic retrocolic (RC) reconstruction, has provided controversial results and the influence of the chosen route of reconstruction is still a matter of discussion.^[14–16]^ Tani et al^[14]^ suggested that the route of gastrojejunostomy (GJ) after a classic Whipple's resection or a duodenojejunostomy (DJ) after a PPPD might be to help to prevent DGE and further revealed that an AC route leads to lower incidences of DGE, as compared with a RC route. However, a RCT conducted by Eshuis et al^[16]^ demonstrated that the AC and RC route of GJ reconstruction after PD does not influence the postoperative incidence of DGE.

Currently, 2 systematic reviews and meta-analyses focused on this topic have been published.^[17,18]^ RC was reported to have higher incidence of DGE than cases associated with AC procedure. However, these previous meta-analyses maybe limited by its small sample sizes; the pooled results of these studies may be unreliable and underpowered for comparison among surgical techniques because potential studies and publication biases are more likely to occur. Therefore, we conducted an update analysis that included the largest available database from RCTs and RSCSs to overcome these limitations, and to investigate the relationship between the AC versus RC route of GJ after PD or DJ after PPPD and the incidence of DGE using the meta-analytical method and sensitivity analyses.

## 2 Materials and methods

### 2.1 The literature review

This study was performed under a human investigational protocol that was approved and monitored by the Institutional Review Board of The first affiliated hospital of Chongqing Medical University. We adhered to the 2009 preferred reporting items for systematic reviews and meta-analysis (PRISMA) statement.^[19]^ An electronic databases search of the Medline Ovid, PubMed, Cochrane Library, and the Controlled Trials Registry was performed, using the following Mesh search headings and their combinations “Pancreaticoduodenectomy,” “Pylorus-preserving pancreaticoduodenectomy,” “Delayed gastric emptying,” “gastrojejunostomy,” “duodenojejunostomy,” articles published in English language as a limit. We gathered all RCTs and RSCSs between 1991 and 2018 and compared the outcomes of AC and RC route of GJ after PD or DJ after PPPD. Retrieval time was ended by December 2017. Title and abstracts of each identified publication were screened, and only publications that reported the clinical outcomes of this analysis were further retrieved.

### 2.2 Inclusion and exclusion criteria

Inclusion criteria for all eligible studies must have to compare the outcomes of AC to RC reconstruction after PD or PPPD and it reported the postoperative outcome of incidence of DGE or at least one of the measured outcomes that referred as follows: when 2 studies containing overlapping (more than 50%) patients were reported from the same institution or authors, either the one of higher quality or the most recent publication was included in the analysis unless an older publication had more measured outcomes or an RCT publication; and studies with limited information, cases reports, and results that were not published in English language were excluded from this analysis.

### 2.3 Measured outcomes and definitions

The primary outcome measure was the postoperative incidence of DGE, according to the International Study Group of Pancreatic Surgery (ISGPS) consensus definition,^[20]^ grade A was defined as nasogastric intubation (NGT) lasting more than 3 postoperative of days (POD) or the inability to tolerate a solid diet by POD 7; grade B was defined as NGT lasting for 8 to 14 days, the need for reinsertion of the NGT after 7 days, or the necessity of prolonged gastric drainage and a delayed return to solid food intake; grade C was defined as NGT lasting more than 14 days, the need for NGT reinsertion after 14 days, or the inability to tolerate a solid diet by POD 21 (Table 1).

**Table 1:**
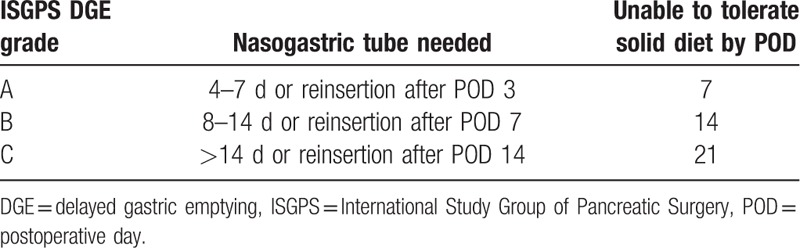
ISGPS definition of DGE grade.

The following secondary outcomes were used to compare AC with RC route:

(1) Operative parameters: operative time (minutes); blood loss (ml), transfusion rate.
(2) Postoperative outcomes: days until to liquid diet (days); days until to solid diet (days); lengths of hospital stay (days).
(3) Postoperative complications: overall complications, pancreatic leakage; bile leakage; wound infections, intra-abdominal abscess, reoperation, and intra-abdominal hemorrhage.
(4) Postoperative mortality.

A pancreatic fistula was defined as drainage of fluid with an amylase concentration 3 times the upper limit of normal serum as per the ISGPF definition.^[20]^

### 2.4 Data extraction and quality assessment

Two authors (QJQ and LM) examined the studies independently and extracted data according to a predefined criteria. If data were not presented in the articles, the corresponding authors were contacted by email to specifically ask for the missing information. If all required numbers were obtained, the study was included. Any discrepancy between the 2 reviewers was assessed and resolved by panel consensus. The methodological quality of each RCT was evaluated by individual components based on the Cochrane risk of bias tool.^[19]^ For RSCSs, the quality of these studies was assessed by using the Modified Newcastle–Ottawa Score,^[21]^ which allocates a maximum of 9 points each to patient selection, the comparability of the 2 groups (AC and RC), and outcome assessment.

### 2.5 Statistical analysis

This meta-analysis was performed in line with recommendations from the Cochrane Collaboration and the Quality of Reporting of Meta-analyses guidelines.^[22,23]^ The statistical software Review Manager version 5.0 (The Cochrane Collaboration, Oxford, United Kingdom) was used to perform all statistical analyses. For the analysis, continuous data with weighted mean differences (WMDs) and corresponding standard deviations were presented as weighted WMDs with 95% confidence intervals (CIs). However, odds ratios (ORs) with 95% CIs as the summary statistics were used to perform statistical analysis for dichotomous variables. Clinical heterogeneity was tested by means of the *I*^2^ value; a value exceeding 50% was considered to represent a significant difference. A random-effects model was used to report the results of heterogeneous data; otherwise, a fixed-effects model was used. Funnel plots were constructed to detect and assess publication bias and any associations between treatment estimates and sample size. Forest plots were constructed, and the value of *P* < .05 was considered to indicate statistical significance.

## 3 Results

### 3.1 Study selection

**Figure 1:**
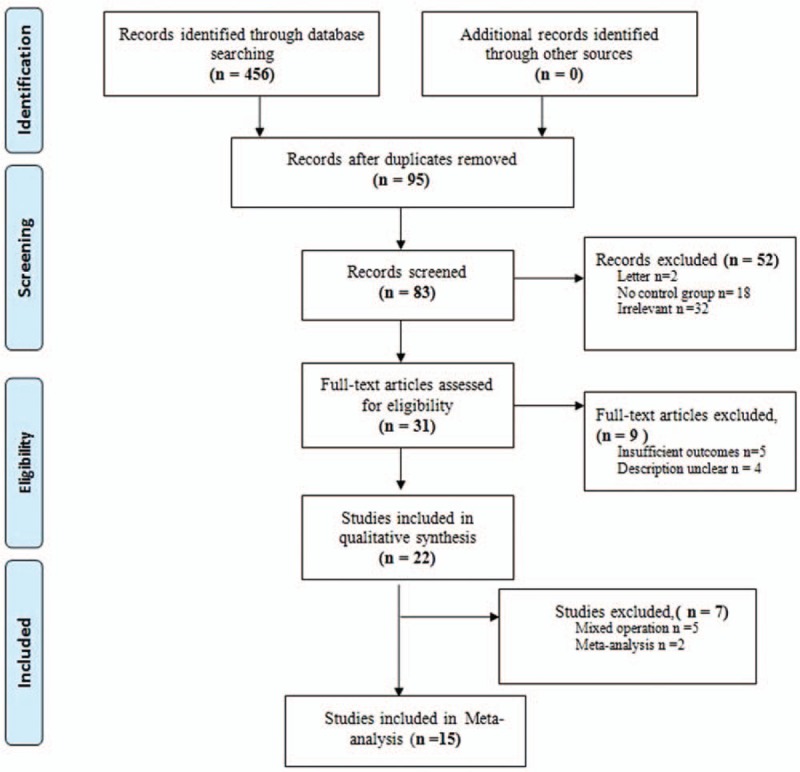
The PRISMA flowchart of literature review. PRISMA = preferred reporting items for systematic reviews and meta-analysis.

The PRISMA flow chart of literature search strategies is illustrated in Figure 1. Initially, a total of 456 potential articles published until 2017 were identified from literature searches after screening all titles and abstracts; no other eligible studies were found from other sources. Overall, 31 articles were included for a full-text evaluation. Of these, 7 were excluded for not meeting the inclusion criteria. In addition, 4 authors were contacted for additional information or to correct inaccurate information, and 2 provided data that were incomplete or not extractable from the original report. Subsequently, 22 studies were potentially included in this study. Of these, 5 studies, in which mixed groups of surgeries were reported and from which data could not be extracted separately and 2 meta-analysis studies were excluded. Finally, in total, 15^[14,16,24–36]^ studies met our inclusion criteria and were retrieved for more detailed evaluation.

### 3.2 Study characteristics and methodological quality assessment

The baseline characteristics of the 15 included studies and the study design are summarized in Table 2. The sample size of these studies ranged from 30 to 800 patients. Analysis was performed on 2270 patients, of whom 1080 (47.6%) underwent AC route reconstruction and 1190 (52.4%) underwent RC route. The quality assessment of 7 RCTs^[14,16,29,31,32,33,34]^ is shown in Table 3. The quality assessment of RSCSs^[24–28,30,35,36]^ is also presented in Table 2. In general, 6 studies^[24,26,28,30,33,36]^ were considered to be of high quality by achieving a score of 6.

**Table 2:**
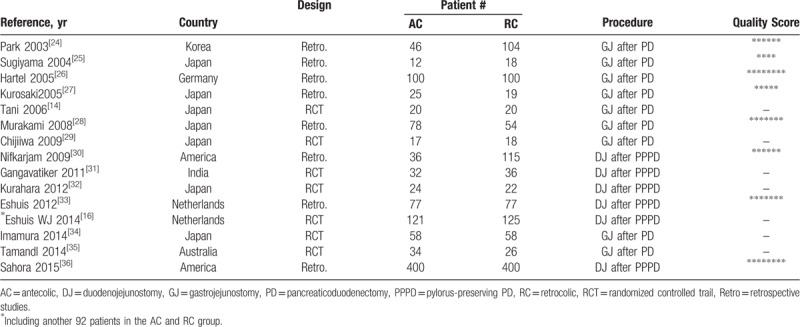
Characteristics of all included studies and quality assessment of retrospective comparative studies (list by publication year).

**Table 3:**
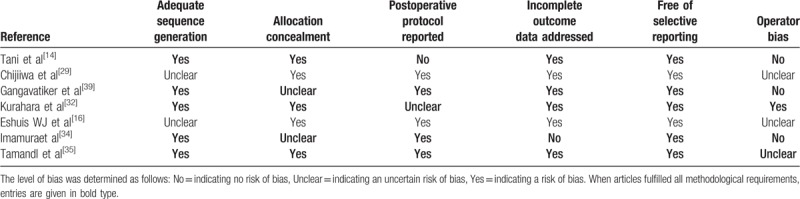
Quality assessment of all eligible randomized controlled trials based on the cochrane risk of bias tool.

### 3.3 Primary outcome measure

**Figure 2:**
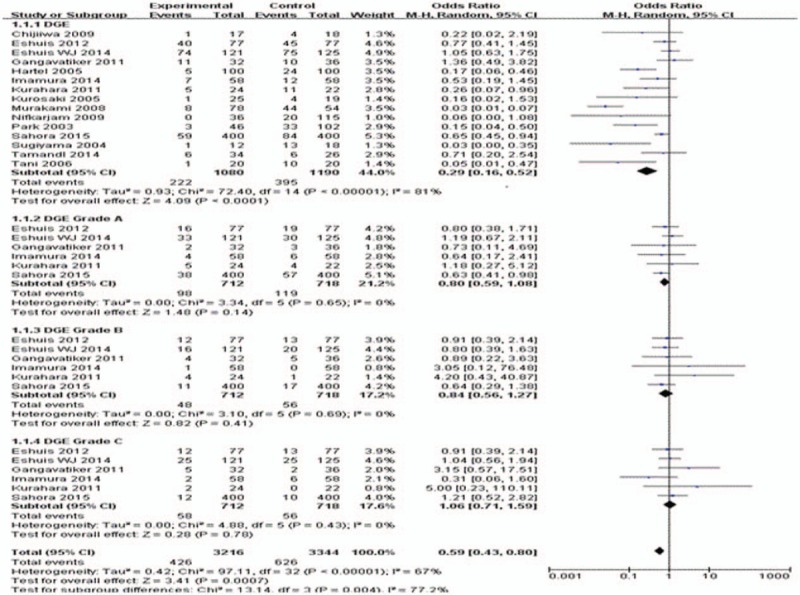
Meta-analysis of delayed gastric emptying and subgroup analysis of Grade A, B, and C.

Basic demographics and treatment characteristics did not differ among the study populations. The overall observed incidence of DGE in the 2270 analyzed patients was 27.2% (Table 4). There was a significant difference in the overall incidence of DGE between the AC and the RC group (OR = 0.29; 95% CI, 0.16–0.52; *P* < .0001; Fig. 2). However, no significant differences were seen in any grade A (*P* = .14), grade B (*P* = .41), and grade C (*P* = .78) DGE between AC and RC reconstruction by subgroup analysis, respectively.

**Table 4:**
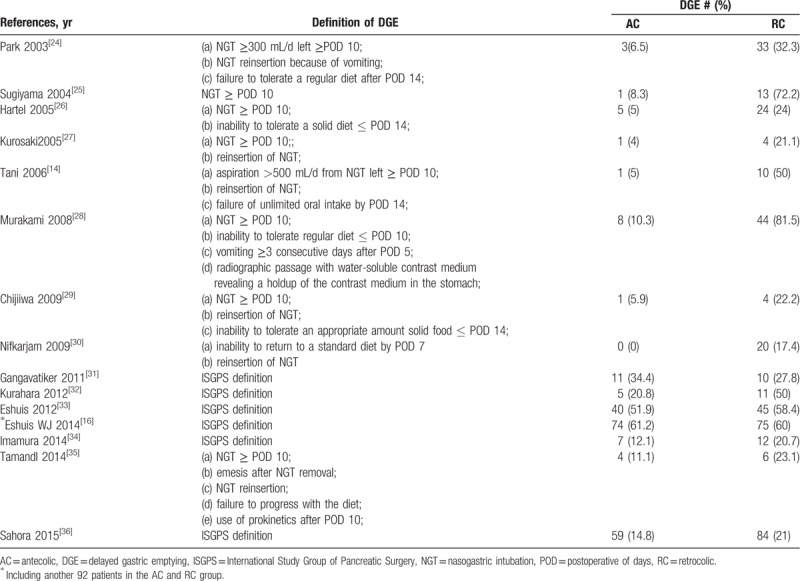
Diverse definitions of DGE.

### 3.4 Secondary outcome measures

#### 3.4.1 Meta-analysis of operative parameters (Fig. 3)

Twelve studies^[14,16,25,27–32,34–36]^ reported data on operative time that included a total of 1704 patients. The meta-analysis showed that operative time did not differ significantly between the 2 operations (MD = −0.94; 95% CI, −9 to 7.11; *P* = .82). Similarly, the estimated blood loss (MD = −46.11; 95% CI, −179.18 to 86.97; *P* = .5) and transfusion rate (OR = 0.76; 95% CI, 0.12 to 5.07; *P* = .78) were also not differ significantly, although these findings were associated with significant heterogeneity between the studies (*I*^2^ = 81% and 89%).

**Figure 3:**
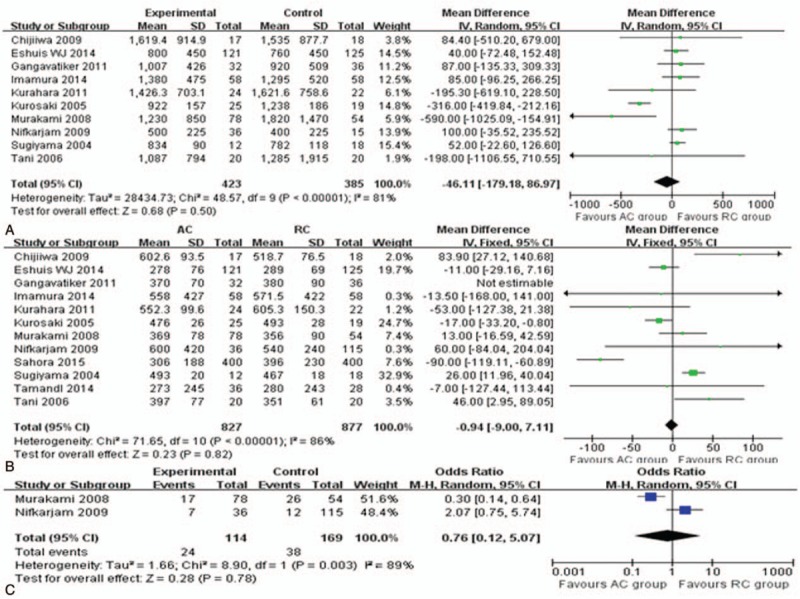
Meta-analysis of operative parameters: (A) operative time, (B) blood loss, (C) transfusion rate.

#### 3.4.2 Meta-analysis of postoperative outcomes (Fig. 4)

The length of hospital stay was measured in 14 studies^[14,16,24–26,28–36]^ including 2228 participants, meta-analysis performed for which showed that patients undergoing AC reconstruction were found to have a significantly shorter than those undergoing RC procedure with a difference of 3.29 days (95% CI, −5.2 to −1.39; *P* = .0007). However, there was significant heterogeneity between the 14 studies reporting on this outcome (HG *P* < .00001). The time until to liquid and solid diet were both found to be significantly earlier in the AC group by 1.12 days (95% CI, −1.77 to −0.48; *P* = .0006) and by 0.71 days (95% CI, −0.88 to −0.54; *P* < .0001) when compared with the RC group, respectively.

**Figure 4:**
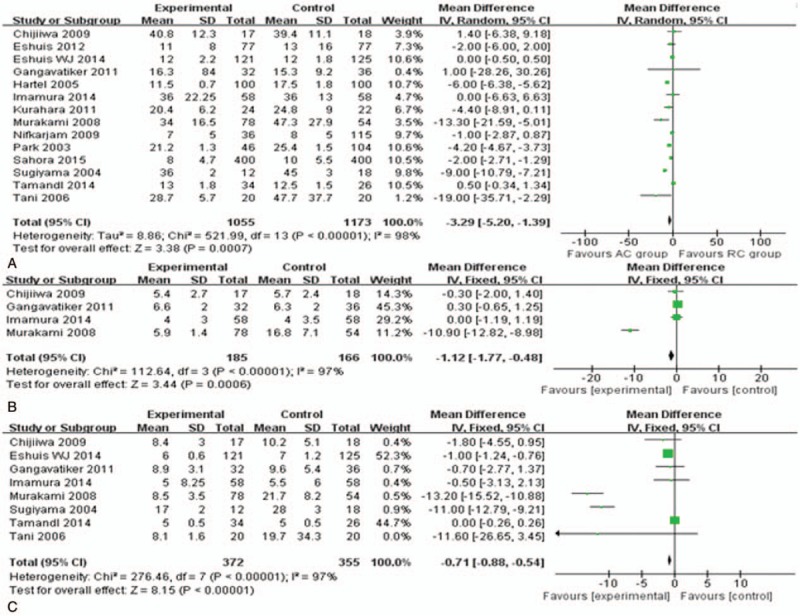
Meta-analysis of postoperative parameters: (A) hospital stay, (B) days until to liquid diet, (C) days until to solid diet.

#### 3.4.3 Meta-analysis of postoperative morbidity

**Figure 5:**
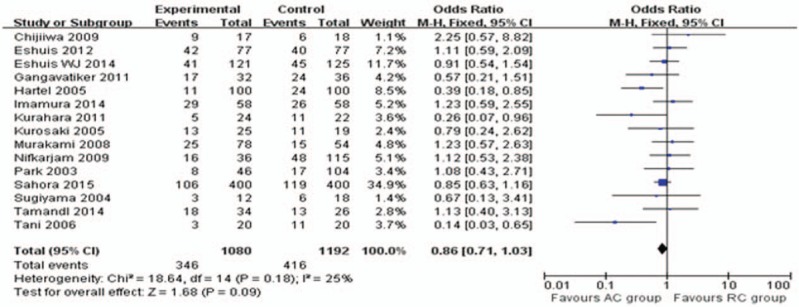
Meta-analysis of overall morbidity.

**Figure 6:**
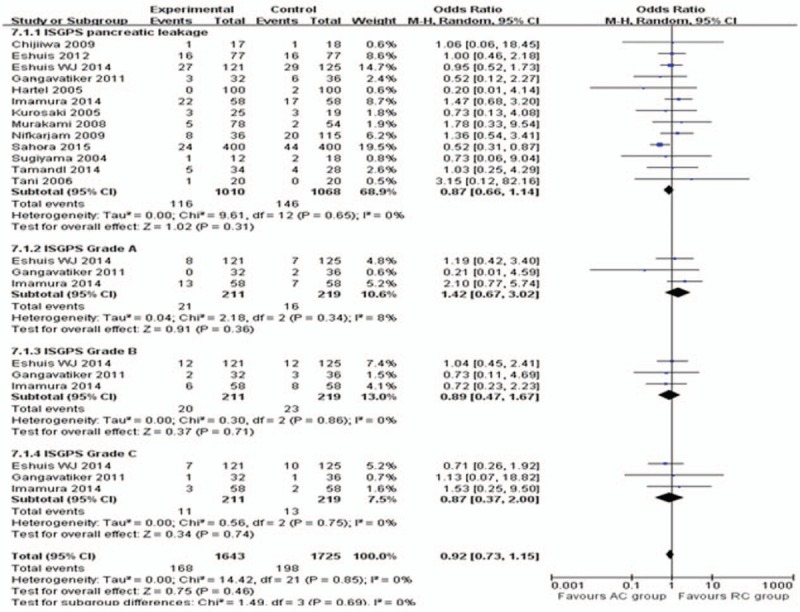
Meta-analysis of pancreatic fistula and subgroup analysis of Grade A, B, and C.

All included studies reported on the overall incidence of postoperative morbidity. The meta-analysis showed that the incidence of overall morbidity was 0.86% lower in the AC group than in the RC group, although this difference was not statistically significant (95% CI, 0.71–1.03; *P* = .09; Fig. 5). Data on postoperative incidence of pancreatic fistula were available in 13 out of 15 studies.^[14,16,25–31,33–36]^ Pooled analysis showed a fewer pancreatic fistula rate in the AC group without reaching the level of statistical significance (OR = 0.87; 95% CI, 0.66–1.14; *P* = .31; Fig. 6), as well as the subgroup analysis of ISGPS grade A (*P* = .36), B (*P* = .71), and C (*P* = .74), respectively. Seven studies^[14,16,27,29,31,32,34]^ reported on the wound infection rate after surgery. Meta-analysis showed there was no significant difference between the AC and RC groups (OR = 0.81; 95% CI, 0.55–1.2; *P* = .3), as were the rates of reoperation (OR = 0.54; 95% CI, 0.28–1.07; *P* = .08). The incidence of bile leakage was measured in 8 studies^[14,16,27,31–35]^ with 774 participants. Meta-analysis showed there was no significant difference between the AC and RC groups (OR = 1.1; 95% CI, 0.55–2.21; *P* = .78), as were the incidence of intra-abdominal abscess and hemorrhage (OR = 1.72; 95% CI, 0.76–3.88, *P* = .19) from 12 studies^[14,16,25,27–33,35,36]^ and 6 studies,^[29–31,33–35]^ respectively.

#### 3.4.4 Meta-analysis of mortality

**Figure 7:**
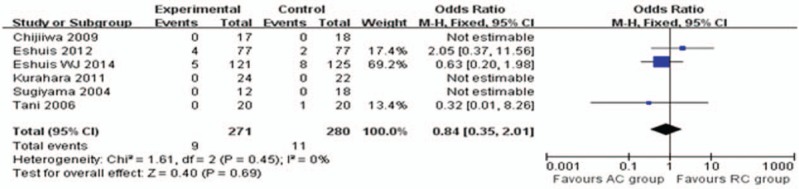
Meta-analysis of mortality.

As related to postoperative mortality, there were only 6 studies^[14,16,25,29,32,33]^ reporting and suggesting that there was no significant difference between the 2 groups by pooled analysis (OR, 0.84; 95% CI, 0.35–2.01; *P* = .69; Fig. 7).

### 3.5 Sensitivity analysis

The sensitivity analysis included the following:

(1) 7 RCTs;
(2) 8 RSCSs;
(3) 13 studies of high quality (7 RCTs and 6 RSCSs with quality score of 6 or more using the modified Newcastle–Ottawa scale);
(4) AC versus RC route of GJ after PD procedure;
(5) AC versus RC route of DJ after PPPD procedure;
(6) 9 studies with more than 30 patients in each group; and
(7) 6 studies with DGE was defined as ISGPS consensus definition.

The results from sensitivity analysis were summarized in Table 5. The forest plots of DGE by sensitivity analysis were illustrated in Figure S1–7.

**Table 5:**
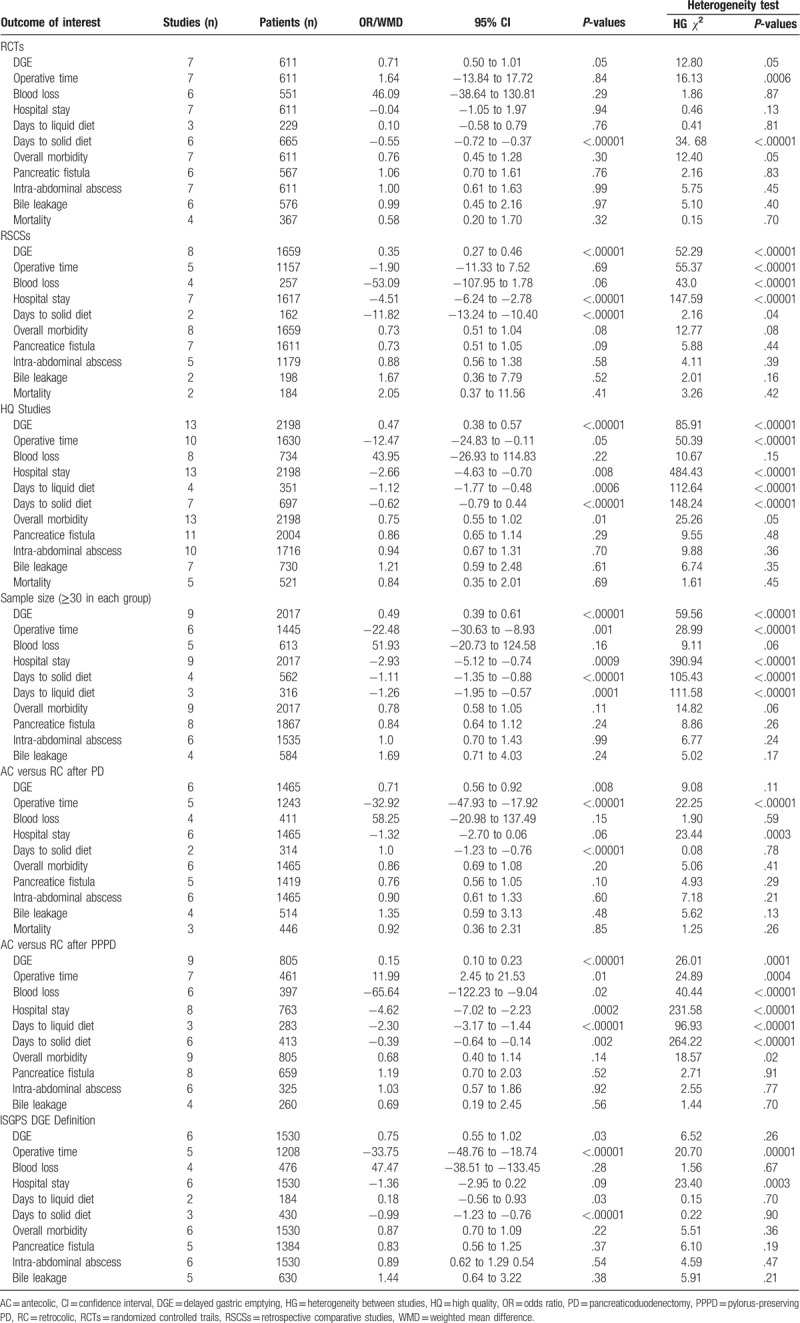
Sensitivity analysis comparing AC versus RC group.

### 3.6 RCTs

When only RCTs^[14,16,29,31,32,33,34]^ were analyzed, DGE rate was still significantly fewer in the AC group by 0.71% (95% CI, 0.50–1.01; *P* = .05). Hospital stay and days until to liquid diet became not significantly between the AC and RC group. The rest of the outcomes were consistent with the overall analysis, including operative time (*P* = .84), blood loss (*P* = .29), overall complications (*P* = .30), incidence of pancreatic fistula (*P* = .76), bile leakage (*P* = .97), and mortality (*P* = .32). There was generally a reduction in the degree of heterogeneity.

### 3.7 RSCSs

Eight RSCSs^[24–28,30,35,36]^ compared the perioperative parameters between the AC and RC groups. Incidence of DGE remained significantly fewer in the AC group by 0.35% (95% CI, 0.27–0.46; *P* < .00001). The other variables remained similar to the original AC versus RC analysis. Heterogeneity was once again reduced.

### 3.8 High-quality studies (scores of >6 and RCTs)

The outcomes showed similar results as the original analysis. The heterogeneity was once again reduced.

### 3.9 Sample size (studies with more than 30 patients in each group)

There were 9 studies^[16,24,26,28,30,31,33,34,36]^ with more than 30 patients in each AC and RC group. Operative time became significantly shorter in the AC group than RC group (MD = −22.48 minutes; 95% CI, − 30.63 to −8.93 minutes; *P* = **.001**). The remaining results were similar to the original analysis, and heterogeneity was once again reduced.

### 3.10 AC versus RC route of GJ after PD procedure

There were 6 studies^[16,30–33,36]^ comparing AC with RC route of GJ after PD procedure. Hospital stay became no significant (MD = 1.32 days; 95% CI, − 2.7 to 0.06 days; *P* = .06), whereas operative time became significantly shorter in the AC group than RC group (MD = 32.92 minutes; 95% CI, − 47.93 to − 17.92 minutes; *P* < .00001). The remaining results were similar to the original analysis, and heterogeneity was once again reduced.

### 3.11 AC versus RC route of DJ after PPPD procedure

There were 9 studies^[14,24–29,34,35]^ comparing AC with RC route of DJ after PPPD procedure. Operative time and estimated blood loss became significantly lower in the AC group than RC group (*P* = .01 and *P* = .02), respectively. The remaining results were similar to the original analysis, and heterogeneity was once again reduced.

### 3.12 ISGPS DGE definition

Six studies^[16,31–34,36]^ reported the overall incidence of DGE according to the ISGPS definition. Incidence of DGE remained significantly fewer in the AC group by 0.73% (95% CI, 0.56–0.97; *P* = .03). Operative time became significantly shorter in the AC group than RC group (MD = −33.75 minutes; 95% CI, − 48.76 to −18.74 minutes; *P* < .00001). The remaining results were similar to the original analysis, and heterogeneity was once again reduced.

### 3.13 Publication bias

**Figure 8:**
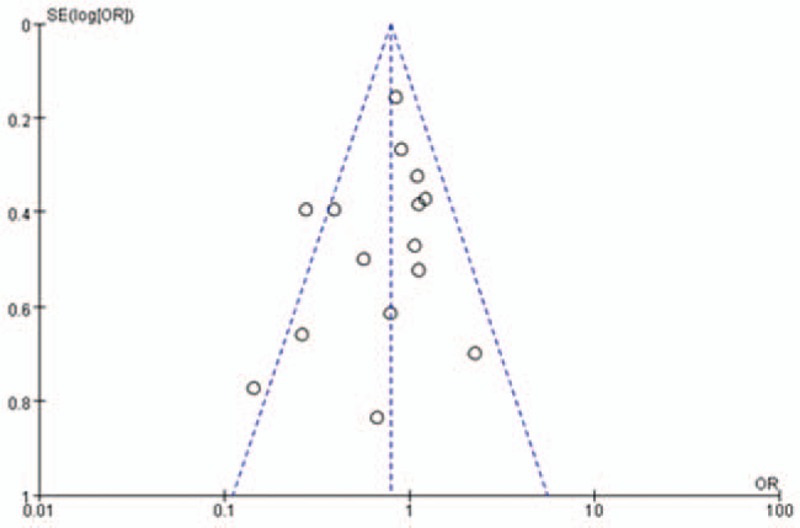
Funnel plot of the incidence of delayed gastric emptying in included studies, showing no publication bias. OR = odds ratio, SE = standard error.

The funnel plot of this study based on postoperative incidence of DGE is shown in Figure 8. All studies except 2 lay inside the limits of the 95% CIs and distributed more evenly about the vertical, showing no evidence of publication bias.

## 4 Discussion

### 4.1 Research frontiers and summary of the study

Since the first report of PPPD was published in 1978,^[37]^ there has a boom in the number of PPPD being performed during the past few years. Several studies have shown that PPPD had a higher incidence of DGE versus the standard PD.^[38–40]^ At the same time, other studies have revealed no difference in the incidence of DGE after PPPD or PD.^[41–43]^ Regardless of the type of PD, DGE is still a frustrating complication following PD or PPPD and is seen in a significant proportion of patients leading to prolonged hospital stay, decreased patient comfort, increased morbidity, and medical costs.^[42,43]^ Many interventions have been tried in an attempt to reduce the high incidence of DGE. One of the most commonly advocated techniques is to perform an AC route of GJ/DJ instead of a RC reconstruction.^[16–18]^ Murakami et al^[28]^ showed that AC reconstruction was better than RC reconstruction in terms of DGE. Another 2 randomized studies also found a significant benefit in favor of AC reconstruction following PPPD.^[14,29]^ However, the influence of the chosen route of reconstruction is still controversial. Therefore, the aim of this study was to compare the incidence of DGE and other parameters between AC and RC route of reconstruction after PD or PPPD using meta-analytical techniques. AC reconstruction was associated with a lower incidence of DGE, shorter hospital stay, and faster recovery to regular diet compared to that in RC group. No statistically significant differences were found between the 2 groups regarding operative time, incidence of pancreatic and bile leakage, and postoperative mortality. These results are similar to majority of the previous published studies.^[14,17,18,28,29]^ Due to the included number of RCTs was too small to make confirm conclusion, we performed several sensitivity analyses including all the RCTs and the high-quality nonrandomized trials and so on, which greatly increase the credibility of the results without necessarily compromising the quality of the study. Results from sensitivity analysis of high-quality and large trials are in accordance with original analysis, which suggesting the reliability of our results.

### 4.2 DGE consensus definition and its influencing factors

The reported incidence of DGE after surgical procedure varies between 6% to 81.5% from the available literature database.^[9–13,16,28,44]^ The lack of a uniform definition of DGE is largely responsible for the diversity. Some authors have defined DGE as requiring NGT decompression for more than 7 or 10 days postoperatively.^[45–47]^ Others have defined DGE as the inability to tolerate a normal diet after the POD 10 or POD 14,^[48,49]^ or a liquid diet after the 7th postoperative day.^[14,50]^ It seems that to establish an internationally accepted uniform definition of DGE is imminent, therefore, a consensus definitions for DGE and other major pancreas-specific complications was proposed by the ISGPS in 2007,^[51]^ which defined the DGE was based on whether there is a need for nasogastric tube placement (and if so, for how long), how soon after the operation the patient is able to tolerate solid oral intake, and whether prokinetic therapy is administered.^[19,52]^ Welsch et al^[13]^ evaluated the ISGPS definition of DGE after PD in a high-volume center and revealed that the ISGPS DGE definition is feasible and applicable in patients with an uneventful postoperative course.

Although the ISGPS grading system for DGE is clearly measurable, it does not explain why DGE has occurred. Several studies have focused on its possible cause and pharmacologic strategies to reduce the high incidence of DGE have been published so far. Sato et al^[53]^ and other researches^[54–56]^ revealed that DGE have a correlation of intra-abdominal complications such as anastomotic leak or abscess. Gastric dysrhythmias, disruption of gastroduodenal neural connections, ischemia of the pylorus muscle, and ligation of the right gastric artery were all related to DGE, respectively.^[57–59]^ In addition, preoperative diabetes mellitus condition, cholangitis, previous abdominal surgery as well as retromesenteric route of jejunal reconstruction were significantly associated with a higher incidence of DGE.^[25]^ Park et al^[24]^ found that the incidence of DGE was 31.7% in the RC group, but only 6.5% in the AC group. Similay results were found by Sugiyama et al,^[25]^ which reported that DGE occurred in 8% patients (8%) in the AC group, but in 72% patients in the RC group. Hartel et al^[26]^ reported an incidence of 5% with the AC route and 24% with the RC route. Meta-analysis of these studies has suggested that the incidence of DGE is lower with the AC route than with the RC route. In the future, to research the exact mechanism of DGE will become more and more urgent and important.

### 4.3 Limitations and recommendations

This systematic review and meta-analysis still have several limitations that must be taken into account. First, the majority of our included studies were comparative studies, although there is evidence that estimates derived from high-quality nonrandomized comparative studies may be similar to those derived from randomized studies. Second, the strength and limitations of meta-analytic techniques have been a source of considerable debate. Third, despite a relatively large number of patients were included in present study, but the sample size was still too small to allow firm conclusions. In future, it is necessary to conduct randomized trials using standardized unbiased methods and well-matched controls.

## 5 Conclusion

This is the very first and largest meta-analysis including RCTs and RSCSs comparing the incidence of DGE and other perioperative outcomes between AC and RC route of GJ after PD or DJ after PPPD. In this meta-analysis, AC route of GJ or DJ reconstruction shows a significantly lower incidence of DGE compared to the RC technique. Further prospective controlled studies are needed for a more comprehensive study between the 2 procedures in future.

## Acknowledgments

The authors thank the staffs and colleagues in the Chinese Cochrane Centre for their help and support. This research was supported by Basic and Advanced Research Project of Science and Technology Commission of Chongqing Municipality (No. cstc2018jcyjAX0825).

## Author contributions

**Methodology:** Cheng Du.

**Software:** Ming Li.

**Writing – original draft:** Jian Qiu, Ming Li.

**Writing – review and editing:** Jian Qiu, Ming Li.

## Supplementary Material

Supplemental Digital Content

## References

[1] Gouma DJ Nieveen van Dijkum EJ Obertop H The standard diagnostic work-up and surgical treatment of pancreatic head tumours. Eur J Surg Oncol 1999;25:113–23. 10218451 10.1053/ejso.1998.0612

[2] Cascinu S Falconi M Valentini V ESMO Guidelines Working Group. Pancreatic cancer: ESMO Clinical Practice Guidelines for diagnosis, treatment and follow-up. Ann Oncol 2010;21:v55–8. 20555103 10.1093/annonc/mdq165

[3] Suzuki S Kaji S Koike N Pancreaticoduodenectomy can be safely performed in the elderly. Surg Today 2013;43:620–4. 23104552 10.1007/s00595-012-0383-6

[4] Kozuschek W Reith HB Waleczek H A comparison of long term results of the standard Whipple procedure and the pylorus preserving pancreatoduodenectomy. J Am Coll Surg 1994;178:443–53. 7909485

[5] Mosca F Giulianotti PC Balestracci T Long-term survival in pancreatic cancer: pyloruspreserving versus Whipple pancreatoduodenectomy. Surgery 1997;122:553–66. 9308613 10.1016/s0039-6060(97)90128-8

[6] Zhou Y Lin L Wu L A case-matched comparison and meta-analysis comparing pylorus-resecting pancreaticoduodenectomy with pylorus-preserving pancreaticoduodenectomy for the incidence of postoperative delayed gastric emptying HPB (Oxford) 2015;17:337–43. 25388024 10.1111/hpb.12358 PMC4368398

[7] Kawai M Tani M Hirono S Pylorus- resecting pancreaticoduodenectomy offers long-term outcomes similar to those of pylorus-preserving pancreaticoduodenectomy: results of a prospective study. World J Surg 2014;38:1476–83. 24370543 10.1007/s00268-013-2420-z

[8] McKay A Mackenzie S Sutherland FR Meta-analysis of pancreaticojejunostomy versus pancreaticogastrostomy reconstruction after pancreaticoduodenectomy. Br J Surg 2006;93:929–36. 16845693 10.1002/bjs.5407

[9] Warshaw AL Torchiana DL Delayed gastric emptying after pyloruspreserving pancreaticoduodenectomy. Surg Gynecol obstetrics 1985;160:1–64. 3964962

[10] Bassi C Falconi M Salvia R Management of complications after pancreaticoduodenectomy in a high volume centre: results on 150 consecutive patients. Dig Surg 2001;18:453–7. 11799295 10.1159/000050193

[11] Yeo CJ Cameron JL Sohn TA Six hundred fifty consecutive pancreaticoduodenectomies in the 1990s: pathology, complications, and outcomes. Ann Surg 1997;226:248–57. 9339931 10.1097/00000658-199709000-00004 PMC1191017

[12] Balcom JH Rattner DW Warshaw AL Ten-year experience with 733 pancreatic resections: changing indications, older patients, and decreasing length of hospitalization. Arch Surg 2001;136:391–8. 11296108 10.1001/archsurg.136.4.391

[13] Welsch T Borm M Degrate L Evaluation of the International Study Group of Pancreatic Surgery definition of delayed gastric emptying after pancreatoduodenectomy in a high-volume centre. Br J Surg 2010;97:1043–50. 20632270 10.1002/bjs.7071

[14] Tani M Terasawa H Kawai M Improvement of delayed gastric emptying in pylorus-preserving pancreaticoduodenectomy: results of a prospective, randomized, controlled trial. Ann Surg 2006;243:316–20. 16495694 10.1097/01.sla.0000201479.84934.ca PMC1448934

[15] Horstmann O Karmus PM Ghadimi MB Pylorus preservation has no impact on delayed gastric emptying after pancreatic head resection. Pancreas 2004;28:69–74. 14707733 10.1097/00006676-200401000-00011

[16] Eshuis WJ van Eijck CH Gerhards MF Antecolic versus retrocolic route of the gastroenteric anastomosis after pancreatoduodenectomy: a randomized controlled trial. Ann Surg 2014;259:45–51. 24096769 10.1097/SLA.0b013e3182a6f529

[17] Su AP Cao SS Zhang Y Does antecolic reconstruction for duodenojejunostomy improve delayed gastric emptying after pylorus-preserving pancreaticoduodenectomy? A systematic review and meta-analysis. World J Gastroenterol 2012;18:6315–23. 23180954 10.3748/wjg.v18.i43.6315 PMC3501782

[18] Bell R Pandanaboyana S Shah N Meta-analysis of antecolic versus retrocolic gastric reconstruction after a pylorus-preservingpancreatoduodenectomy HPB (Oxford) 2015;17:202–8. 25267428 10.1111/hpb.12344 PMC4333780

[19] Moher D Liberati A Tetzlaff J Reprint–preferred reporting items for systematic reviews and meta-analyses: the PRISMA statement. Phys Ther 2009;89:873–80. 19723669

[20] Wente MN Bassi C Dervenis C Delayed gastric emptying (DGE) after pancreatic surgery: a suggested definition by the International Study Group of Pancreatic Surgery (ISGPS). Surgery 2007;142:761–8. 17981197 10.1016/j.surg.2007.05.005

[21] Athanasiou T Al-Ruzzeh S Kumar P Off-pump myocardial revascularization is associated with less incidence of stroke in elderly patients. Ann Thorac Surg 2004;77:745–53. 14759484 10.1016/j.athoracsur.2003.07.002

[22] Clarke M Horton R Bringing it all together: Lancet-Cochrane collaborate on systematic reviews. Lancet 2001;357:1728. 10.1016/S0140-6736(00)04934-5 11403806

[23] Stroup DF Berlin JA Morton SC Meta-analysis of observational studies in epidemiology: a proposal for reporting. Meta-analysis of Observational Studies in Epidemiology (MOOSE) group. JAMA 2000;283:2008–12. 10789670 10.1001/jama.283.15.2008

[24] Park YC Lim SW Jang JY Factors influencing delayed gastric emptying after pylorus-preserving pancreatoduodenectomy. J Am Coll Surg 2003;196:859–65. 12788421 10.1016/S1072-7515(03)00127-3

[25] Sugiyama M Abe N Ueki H A new reconstruction method for preventing delayed gastricemptying after pylorus-preservingpancreatoduodenectomy. Am J Surg 2004;187:743–6. 15191869 10.1016/j.amjsurg.2003.10.013

[26] Hartel M Wente MN Hinz U Effect of antecolic reconstruction on delayed gastricempting after the pylorus-preserving Whipple procedure. Arch Surg 2005;140:1094–9. 16301447 10.1001/archsurg.140.11.1094

[27] Kurosaki I Hatakeyama K Clinical and surgical factors in-fluencing delayed gastric emptying after pyloric-preserving pancreaticoduodenectomy. Hepatogastroenterology 2005;52:143–8. 15783015

[28] Murakami Y Uemura K Sudo T An antecolic Roux en Y type reconstruction decreased delayed gastric emptying after pylorus preserving pancreatoduodenectomy. J Gastrointest Surg 2008;12:1081–6. 18256885 10.1007/s11605-008-0483-1

[29] Chijiiwa K Imamura N Ohuchida J Prospective randomized controlled study of gastric emptying assessed by ^13^C-acetate breath test after pylorus-preserving pancreatoduodenectomy: comparison between antecolic and vertical retrocolicduodenojejunostomy. J Hepatobiliary Pancreat Surg 2009;16:49–55. 19083149 10.1007/s00534-008-0004-3

[30] Nifkarjam M Kimchi ET Gusani NJ A reduction in delayed gastric emptying by classic pancreatoduodenectomy with an antecolic gastrojejunal anastomosis and a retrogastric omental patch. J Gastrointest Surg 2009;13:1674–82. 19548039 10.1007/s11605-009-0944-1

[31] Gangavatiker R Pal S Javed A Effect of antecolic or retrocolic reconstruction of the gastro/duodenojejunostomy on delayed gastric emptying after pancreatoduodenectomy: a randomized controlled trial. J Gastrointest Surg 2011;15:843–52. 21409601 10.1007/s11605-011-1480-3

[32] Kurahara H Shinchi H Maemura K Delayed gastric emptying after pancreatoduodenectomy. J Surg Res 2011;171:e187–92. 22001182 10.1016/j.jss.2011.08.002

[33] Eshuis WJ van Dalen JW Busch ORC Route of gastroenteric reconstruction in pancreatoduodenectomy and delayed gastric emptying. HPB 2012;14:54–9. 22151452 10.1111/j.1477-2574.2011.00403.x PMC3252992

[34] Imamura N Chijiiwa K Ohuchida J Prospective randomized clinical trial of a change ingastric emptying and nutritional status after a pylorus-preserving pancreaticoduodenectomy: comparison between an antecolic and a vertical retrocolic duodenojejunostomy. HPB 2014;16:384–94. 23991719 10.1111/hpb.12153 PMC3967891

[35] Tamandl D Sahora K Prucker J Impact of the reconstruction method on delayed gastric emptying after pylorus-preserving pancreaticoduodenectomy: a prospective randomized study. World J Surg 2014;38:465–75. 24121364 10.1007/s00268-013-2274-4

[36] Sahora K Morales-Oyarvide V Thayer SP The effect of antecolic versus retrocolic reconstruction on delayed gastric emptying after classic non–pylorus-preserving pancreaticoduodenectomy. Am J Surg 2015;209:1028–35. 25124295 10.1016/j.amjsurg.2014.04.015

[37] Traverso LW Longmire WP Preservation of the pylorus in pancreatoduodenectomy. Surg Gynecol Obstet 1978;146:959–62. 653575

[38] Büchler MW Friess H Müller MW Randomized trial of duodenum-preserving pancreatic head resection versus pylorus-preserving Whipple in chronic pancreatitis. Am J Surg 1995;169:65–9. 7818000 10.1016/s0002-9610(99)80111-1

[39] Srinarmwong C Luechakiettisak P Prasitvilai W Standard whipple's operation versus pylorus preserving pancreaticoduodenectomy: a randomized controlled trial study. J Med Assoc Thai 2008;91:693–8. 18672634

[40] Lin PW Shan YS Lin YJ Pancreaticoduodenectomy for pancreatic head cancer: PPPD versus Whipple procedure. Hepatogastroenterology 2005;52:1601–4. 16201125

[41] Di Carlo V Zerbi A Balzano G Pylorus-preserving pancreaticoduodenectomy versus conventional whipple operation. World J Surg 1999;23:920–5. 10449821 10.1007/s002689900600

[42] Leichtle SW Kaoutzanis C Mouawad NJ Classic Whipple versus pylorus-preserving pancreaticoduodenectomy in the ACS NSQIP. J Surg Res 2013;183:170–6. 23410660 10.1016/j.jss.2013.01.016

[43] Tran KT Smeenk HG van Eijck CH Pylorus preserving pancreaticoduodenectomy versus standard Whipple procedure: a prospective, randomized, multicenter analysis of 170 patients with pancreatic and periampullary tumors. Ann Surg 2004;240:738–45. 15492552 10.1097/01.sla.0000143248.71964.29 PMC1356476

[44] Park JS Hwang HK Kim JK Clinical validation and risk factors for delayed gastric emptying based on the International Study Group of Pancreatic Surgery (ISGPS) Classification. Surgery 2009;146:882–7. 19744455 10.1016/j.surg.2009.05.012

[45] van Berge Henegouwen MI van Gulik TM DeWit LT Delayed gastric emptying after standard pancreaticoduodenectomy versus pylorus-preserving pancreaticoduodenectomy: an analysis of 200 consecutive patients. J Am Coll Surg 1997;185:373–9. 9328386 10.1016/s1072-7515(97)00078-1

[46] Horstmann O Becker H Post S Is delayed gastric emptying following pancreaticoduodenectomy related to pylorus preservation? Langebecks Arch Surg 1999;384:354–9. 10.1007/s004230050213 10473855

[47] Martignoni ME Friess H Sell F Enteral nutrition prolongs delayed gastric emptying in patients after Whipple resection. Am J Surg 2000;180:18–23. 11036133 10.1016/s0002-9610(00)00418-9

[48] Yeo CJ Barry K Sauter PK Erythromycin accelerates gastric emptying after pancreaticoduodenectomy. Ann Surg 1993;218:229–38. 8103982 10.1097/00000658-199309000-00002 PMC1242953

[49] Zerbi A Balzano G Patuzzo R Comparison between pylorus preserving and Whipple pancreatoduodenectomy. Br J Surg 1995;87:975–9. 10.1002/bjs.1800820738 7648124

[50] Shan YS Tsai ML Chiu NT Reconsideration of delayed gastric emptying in pancreaticoduodenectomy. World J Surg 2005;29:873–80. 15951944 10.1007/s00268-005-7473-1

[51] Bassi C Dervenis C Butturini G International Study Group on Pancreatic Fistula Definition. Postoperative pancreatic fistula: an international study group (ISGPF) definition. Surgery 2005;138:8–13. 16003309 10.1016/j.surg.2005.05.001

[52] Wente MN Veit JA Bassi C Postpancreatectomy hemorrhage (PPH): an International Study Group of Pancreatic Surgery (ISGPS) definition. Surgery 2007;142:20–5. 17629996 10.1016/j.surg.2007.02.001

[53] Sato G Ishizaki Y Yoshimoto J Factors influencing clinically significant delayed gastric emptying after subtotal stomach-preserving pancreatoduodenectomy. World J Surg 2014;38:968–75. 24136719 10.1007/s00268-013-2288-y

[54] Fabre JM Burgel JS Navarro F Delayed gastric emptying after pancreaticoduodenectomy and pancreaticogastrostomy. Eur J Surg 1999;165:560–5. 10433140 10.1080/110241599750006460

[55] Lermite E Pessaux P Brehant O Risk factors of pancreatic fistula and delayed gastric emptying after pancreaticoduodenectomy with pancreaticogastrostomy. J Am Coll Surg 2007;204:588–96. 17382217 10.1016/j.jamcollsurg.2007.01.018

[56] Tanaka M Sarr MG Role of the duodenum in the control of canine gastrointestinal motility. Gastroenterology 1988;94:622–9. 3338632 10.1016/0016-5085(88)90232-6

[57] Matsunaga H Tanaka M Naritomi G Effect of leucine 13-motilin (KW5139) on early gastric stasis after pylorus-preserving pancreatoduodenectomy. Ann Surg 1998;227:507–12. 9563538 10.1097/00000658-199804000-00010 PMC1191305

[58] Niedergethmann M Shang E Farag Soliman M Early and enduring nutritional and functional results of pylorus preservation vs classic Whipple procedure for pancreatic cancer. Langebecks Arch Surg 2006;391:195–202. 10.1007/s00423-005-0015-3 16491403

[59] Fischer CP Hong JC Method of pyloric reconstruction and impact upon delayed gastric emptying and hospital stay after pylorus preserving pancreaticoduodenectomy. J Gastrointest Surg 2006;10:215–9. 16455453 10.1016/j.gassur.2005.07.017

