# Supplementary material for: Antecolic reconstruction is associated with a lower incidence of delayed gastric emptying compared to retrocolic technique after Whipple or pylorus-preserving pancreaticoduodenectomy
Source: Medicine (Baltimore). 2019 Aug 23;98(34):e16663. doi: 10.1097/MD.0000000000016663 (PMC6716732; doi:10.1097/MD.0000000000016663)
Supplement: Supplemental Digital Content [file medi-98-e16663-s001.doc]

**Figure S1-7**

**Sensitivity Analysis of DGE**


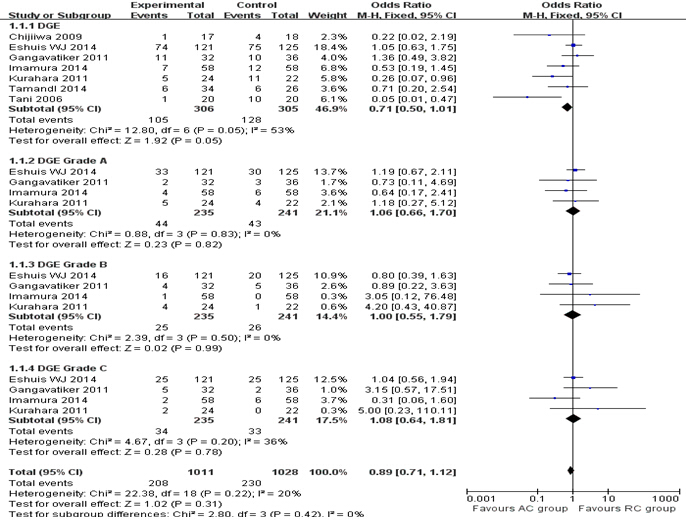


**FIGURE S1.** The forest plots of DGE derived from 7 randomized controlled trails, shows a significantly lower incidence of DGE in the antecolic group.

**
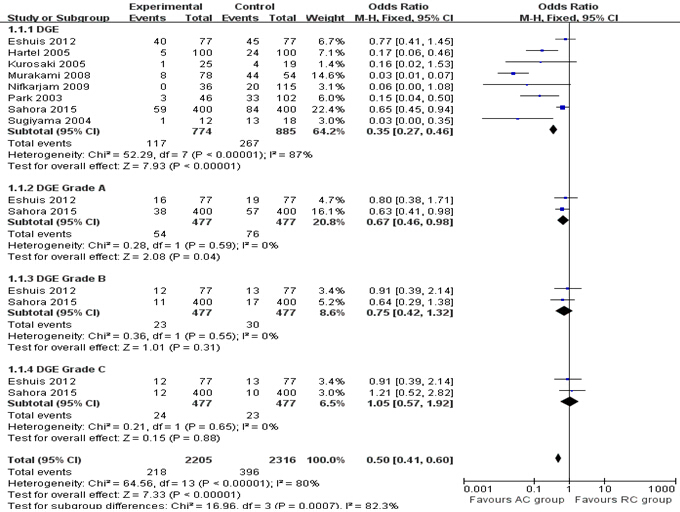
**

**FIGURE S2.** The forest plots of DGE derived from 8 retrospective comparative studies,shows a significantly lower incidence of DGE in the antecolic group.


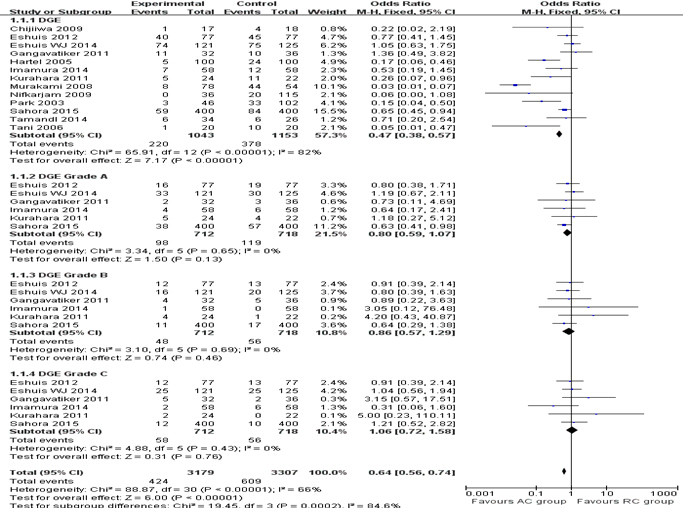


**FIGURE S3.** The forest plots of DGE derived from 13 high quality studies,shows a significantly lower incidence of DGE in the antecolic group.


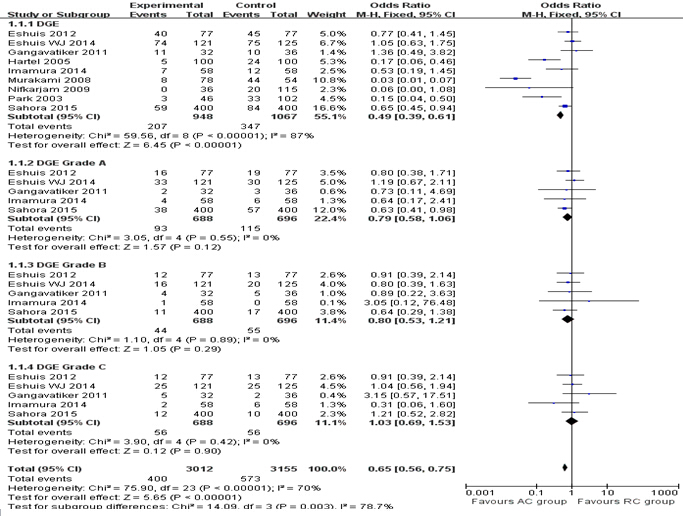


**FIGURE S4.** The forest plots of DGE derived from 9 sdudies with sample size ≥ 30 in each antecolic and retrocolic group, shows a significantly lower incidence of DGE in the antecolic group.

**
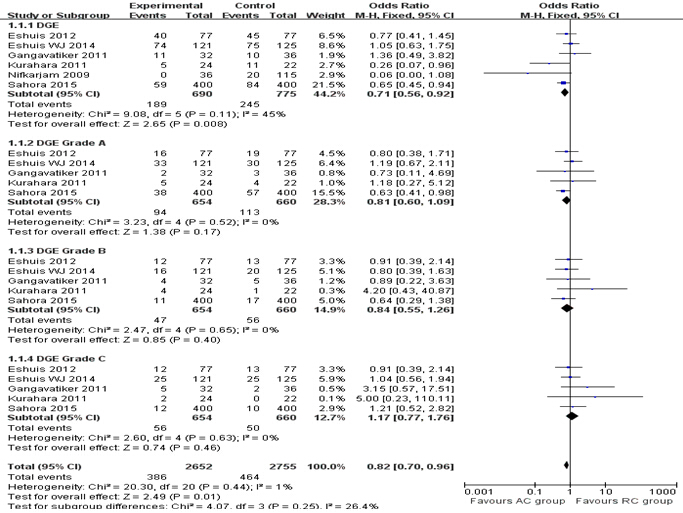
FIGURE S5.** The forest plots of DGE derived from 6 studies with AC vs. RC Route of GJ after PD Procedure, shows a significantly lower incidence of DGE in the antecolic group.


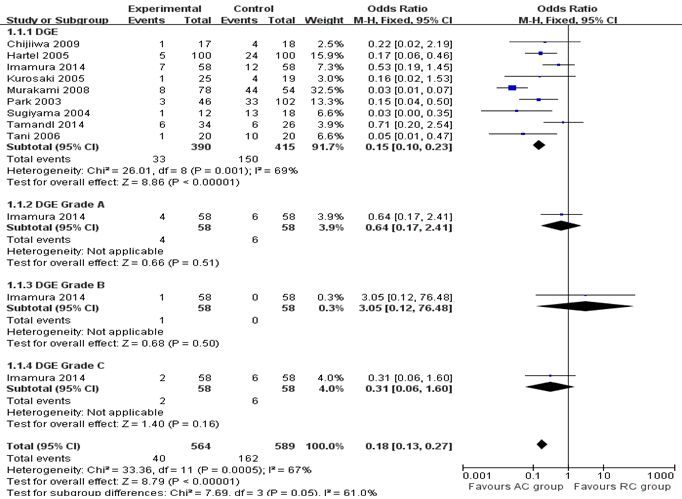


**FIGURE S6.** The forest plots of DGE derived from 9 studies with AC vs. RC route of DJ after PPPD Procedure, shows a significantly lower incidence of DGE in the antecolic group.


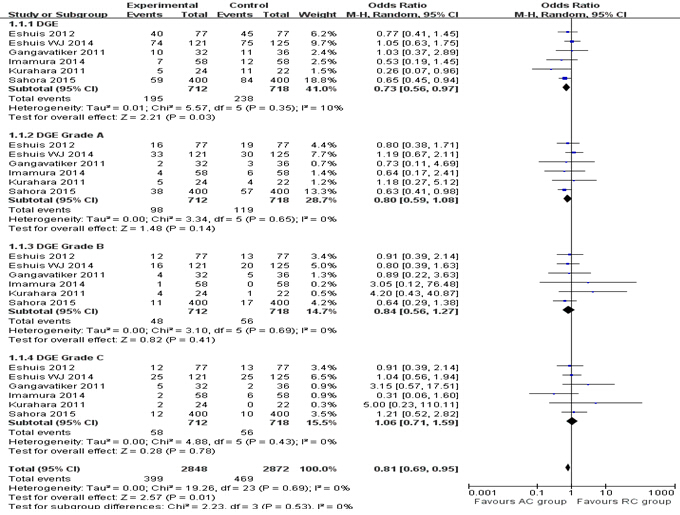


**FIGURE S7.** The forest plots of DGE derived from 6 studies with DGE was defined as ISGPS consensus definition, shows a significantly lower incidence of DGE in the antecolic group.
